# Automated code compliance checking research based on BIM and knowledge graph

**DOI:** 10.1038/s41598-023-34342-1

**Published:** 2023-05-01

**Authors:** Junlong Peng, Xiangjun Liu

**Affiliations:** 1 College of Transportation Engineering, Changsha University of Science and Technology, Changsha, 410114 Hunan China; 2 College of Transportation Engineering, BIM Experimental Center, Changsha University of Science and Technology, Changsha, 410114 Hunan China

**Keywords:** Civil engineering, Computer science

## Abstract

Automated code compliance checking plays an important role in moving the construction industry forward. While traditional drawing review relies on the identification of industry experts, the purpose of this study is to realize automatic code review by using BIM technology and knowledge graph technology. The method is based on knowledge graph to transform the specification provisions involved in the review of drawings into a computer-recognizable structured language using natural language processing technology to form a human–machine-readable knowledge graph pattern. For the review of BIM models, the BIM model information is exported and the global resource description framework of the building model is obtained in protégé, and the review report is finally obtained after mapping rules and review rules. Finally, the feasibility of the method is verified by an example. This study can effectively solve the problems of “manual dependency” and “inefficiency” in the process of review.

## Introduction

**Figure 1:**
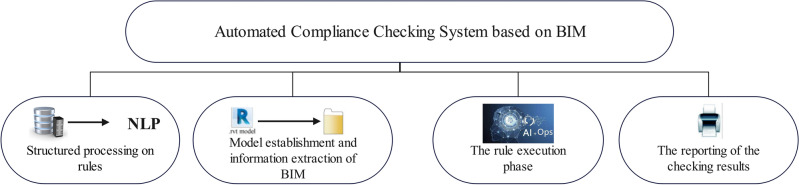
Framework of the BIM review system.

With the second revolution of the construction industry, the development of Building Information Modelling (BIM), the construction industry is gradually changing to the intelligent industry. In recent years, domestic and international vigorously promote the realization of BIM automated review, and the use of BIM technology and Web technology has brought the possibility of automatic code compliance checking. Automatic code compliance checking refers to a computer-based method to automate the drawing review process. The traditional manual drawing review is difficult to meet the requirements of high efficiency and high accuracy. The application of BIM technology in drawing review is conducive to improving the comprehensiveness of drawing review and accelerating the efficiency of drawing review. BIM is the most advanced technology in the field of engineering^1–3^, integrates architectural design, green building, visualization, information integration, schedule cost, etc., and has been applied in actual projects. Regarding the automated review, relevant research results have been obtained. The initial research was originated from the "BP Expert" system proposed by Singapore for checking 2D drawings^4^, later it was gradually upgraded to a review system for BIM models. Ma et al.^5^ designed an algorithm for completing construction quality checkpoints using BIM technology, Xing et al.^6^ analyzed the process framework for the implementation of an automated construction drawing review system. The establishment of the BIM automatic review system can be divided into four steps, as shown in Fig. 1: first, the structured processing of the rules, second, the establishment of the BIM model and information extraction, third, the execution and reasoning of the review rules, and fourth, the output of the review results^7^. The first and second steps are the main obstacles to the implementation of automated code checking.

Different from previous studies, this study added the technique of knowledge graph to the structured processing of the rules in the first step. Knowledge Graph was first proposed by Google in 2012^8^, and its purpose is for users to get a better search experience. Nowadays, knowledge graph technology is used in finance, geography, Internet of Things, healthcare, entertainment and other fields. Its power lies in the semantic processing and interconnected organization capabilities, which provide the basis for intelligent information applications.

## Literature review

At present, compliance checking are primarily carried out by qualified specialists. Given the complexity of engineering, manual compliance checking is time-consuming, costly and error-prone, and automated code checking (ACC) can reduce time and costs and improve the quality of reviews. As web technologies continue to emerge, data can be accessed in a machine-readable manner, allowing some processes to be automated. Extracting requirements from large volumes of written text documents is a major challenge, and natural language processing (NLP) is a bridge between machine language and human language^9^ for the purpose of human–machine communication. Zhang et al.^10^ propose a new automated compliance checking (ACC) system that addresses the problem of manual coding rules. Solihin et al.^11^ proposed a rule classification method for a large number of complex specifications, and Zhong et al.^12^ developed a deep learning model that combines information retrieval with natural language processing (NLP). There are off-the-shelf NLP tools for knowledge acquisition, but improvements to structured text processing tools can be made to better fit the domain features, Zhang et al.^13^ proposed a semantic, rule-based NLP approach for automatically extracting information from construction specification documents.

In addition to working on the complex issues of formalizing rules related to architectural design, another major difficulty that must be addressed in order for an automated code checking procedure to be successful lies in data availability and accessibility. Generally, the IFC standard format is considered well suited for automated compliance checking^14,15^, while Malsane et al.^15^ suggest that BIM models do not usually include the level of detail required for automated code checking. Zhang^16^ et al. argue that the IFC standard format also lacks the information needed for plan review and may be distorted or lost during data exchange, and propose an approach to extend the IFC using natural language processing techniques. In fact, BIM design and code checking as two links, at present, it is almost impossible to expect BIM modelers to design with all review information explicitly defined. Several commercial ACC systems that exist in the market today also require artificially completing specific information. Some studies have shown that machine learning can be effective in achieving semantic enrichment and resolving discrepancies in the exchange of IFC data^17^, but representing the information contained in BIM models in a format suitable for training machine learning algorithms remains a major challenge^18^. Solihin et al.^19^ proposes a novel approach of transforming building data into a simplified schema. Zhong et al.^20^ propose an ontology-based framework that can integrate the information needed by any system.

To sum up, there are preliminary studies in information code review at home and abroad, but in the specification of the text structured method, the scope is relatively single and in the semantic enrichment of the building model, although some of the methods can correct the errors in the export of information^21–23^, but can not imply the applicability of such methods to solve such problems. As a result, it is still difficult to fully implement automated BIM review at this time. However, this study proposes that adding knowledge graph to the automatic code checking system can help to improve the structure of normative text, and the knowledge graph technology has been applied in the field of architecture, such as Fang et al.^24^ using knowledge graph to identify and infer possible hazard sources on construction sites; Zhou et al.^25^ proposed an intelligent fault diagnosis method based on ontology and FMECA (Failure Mode, Effects and Criticality Analysis) to solve the wind turbine failure problem; Cheng et al.^26^ proposed a new method to analyze large-scale building operation data using graph mining technology; Mu et al.^27^ applied knowledge graph to the study of intelligent review of building firefighting plans. However, for the construction industry, the above studies are not enough to implement the widespread application of BIM technology and automatic plan review technology. Using knowledge graph to transform the complicated code provisions into a computer-recognizable language and help the review of drawings to be more efficient and accurate is one of the core contents of this paper, which is of great significance to promote the intelligent development of the construction industry.

## Automated code compliance checking research based on BIM and knowledge graph

### Problem description

**Figure 2:**
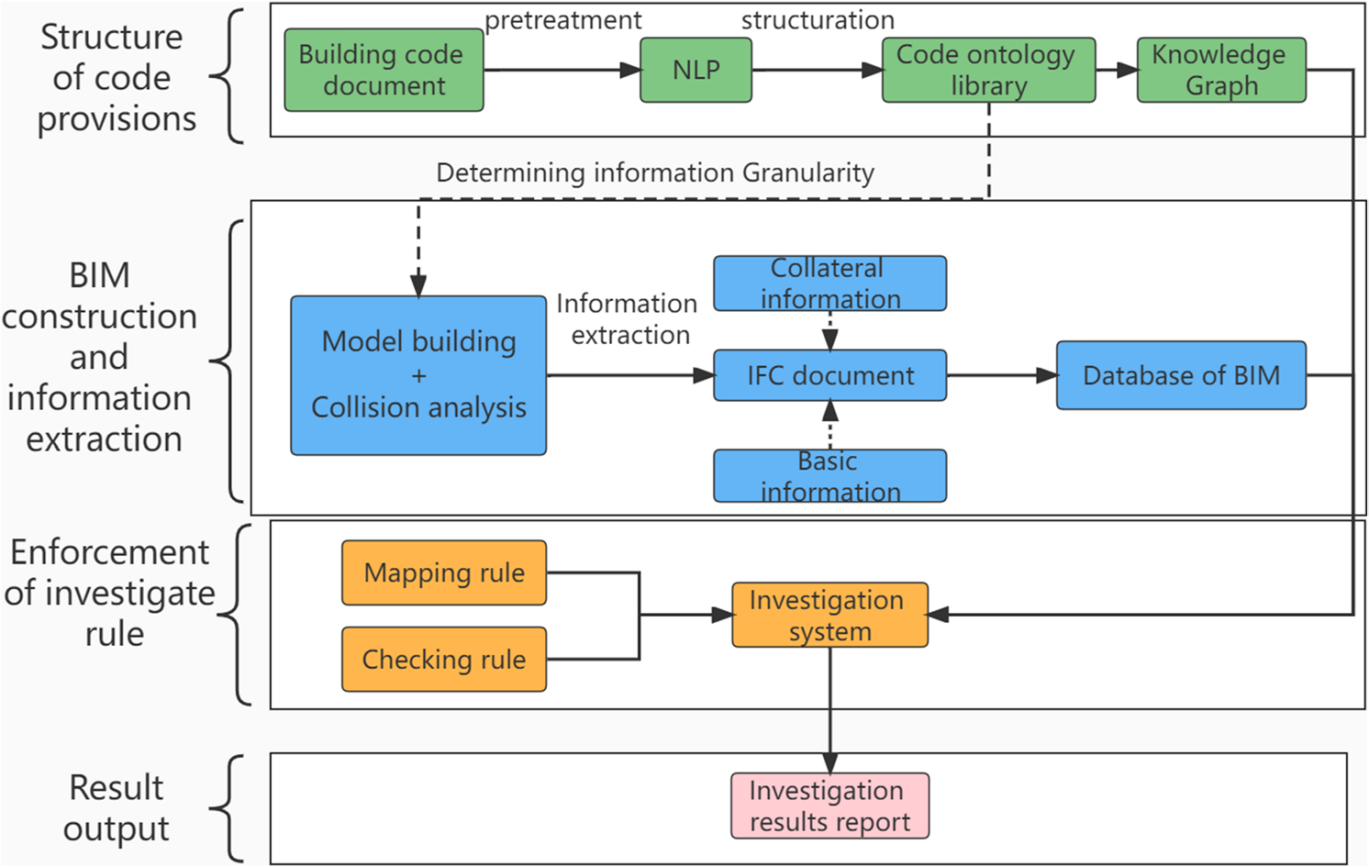
Key technology flow of automated review system based on BIM and knowledge graph.

In view of the manual drawing review is time-consuming, laborious and low efficiency problems, in this study, BIM technology and knowledge graph technology are proposed to promote the full realization of automated code checking. At the same time, the development of automatic review technology can also promote the application of BIM technology and promote the development of the construction industry. In order to realize the whole process automation, this paper will demonstrate from four aspects: standard database, model information export for review, review process, and results output. The technical road map of this paper is shown in Fig. 2.

### Construction of the knowledge graph

#### Code structure

**Figure 3:**
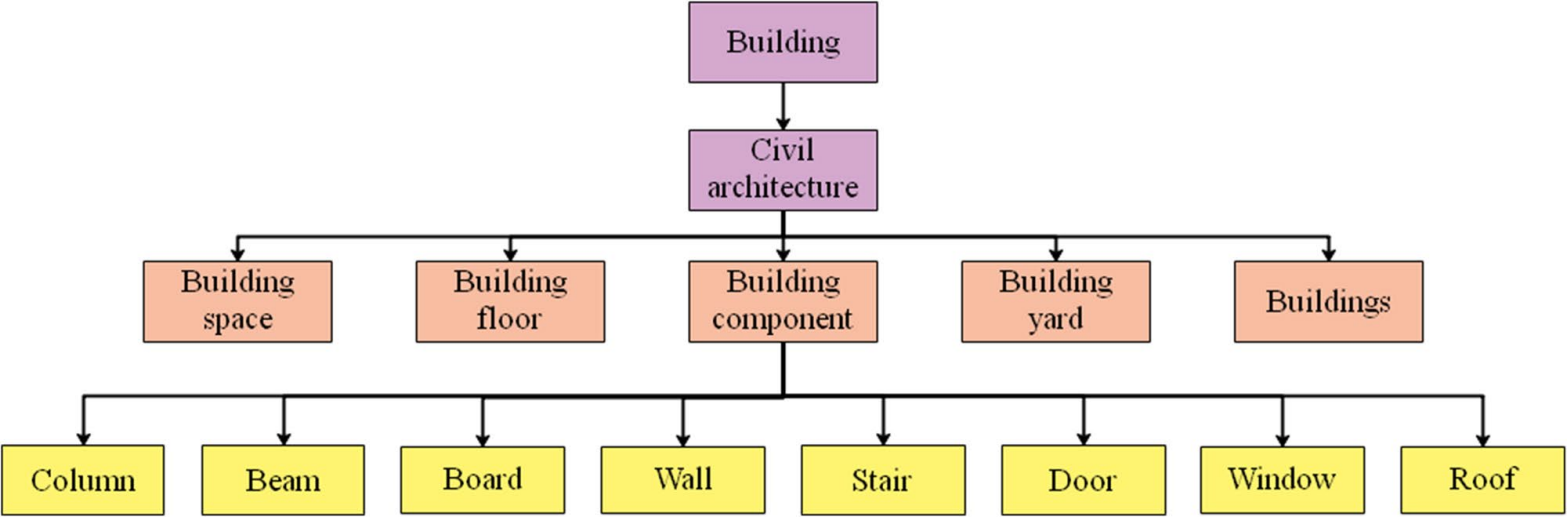
Schema layer.

At present, China’s construction engineering field has published several design code documents, the scope of application of various types of codes is different and there are content references between each other, the content is more scattered, manual processing is difficult, inefficient and error-prone. To solve such problems, this paper constructs a knowledge graph with the mandatory provisions in the 《Uniform Standards for Civil Building Design》, 《Code for fire prevention in building design》, 《Accessibility Design Code》, 《Fire Prevention Code for Building Interior Decoration Design》 and 《Building Water Supply and Drainage Design Code》 as the objects, and the establishment of the knowledge graph is the premise of the review of BIM model. The knowledge graph has a schema layer and a data layer, and the schema layer is mainly for ontology construction, which helps to organize domain knowledge and eliminate the semantic ambiguity of professional terms. According to the domestic and international literature survey study, the more mature methods in structuring the articles are mainly Ontology and context-independent methods. Ontology is defined as an explicit and shared conceptualization of a given domain, which provides explicit logic about three types of things, classes, instances and attributes. For better information integration and extraction, this paper adopts ontology method to classify civil building ontology with reference to IFC standard. As shown in Fig. 3.

**Figure 4:**
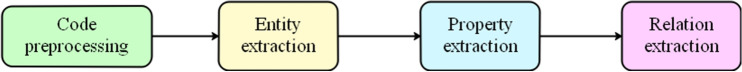
Data layer processing.

The data layer consists mainly of the basic units of the knowledge graph such as entities, property, and relationships, before which we need the following treatment of the specification provisions, as shown in Fig. 4.

As the specification regulations are mostly presented in the form of text or table, the text regulations may point to multiple objects or multiple constraints, and the form of table is difficult to be recognized by computer, so it is necessary to pre-process the specification beforehand. Pre-processing is to remove, clean and normalize the collected data to facilitate subsequent data mining and knowledge extraction. Using the NLPIR Chinese word separation system, information extraction of unstructured and semi-structured data is performed, and the text is divided and set into three main parts: entities, attributes and relations according to its lexical nature. Since the text is a standardized narrative and has no pronoun referents, there is no problem of entity unification and denotative disambiguation.

The specific extraction process we use natural language processing technology and machine learning algorithms, first, collect training data in related fields, which should contain labeled entities, which can be manually labeled or labeled using automatic labeling tools. Next, cleaned and pre-processed the data, such as removing irrelevant information, removing duplicate data, etc. The next step is feature extraction: use natural language processing technology to extract features of text data, such as word vector, character-level features, part of speech annotation, named entity recognition, etc. These features can be used to describe the contextual information and recognition patterns of entities. Model selection and training: Conditional Random Fields (CRF) is used for entity extraction, CRF is a machine learning method commonly used in natural language processing. CRF is a probabilistic graph model, which is used to model sequence annotation problems, such as named entity recognition, word segmentation, part of speech annotation and other tasks. Compared with Hidden Markov Models (HMM), CRF is able to handle long-distance dependencies and non-local features better, and therefore performs better in the sequence annotation task.

The basic idea of CRF is that given the input sequence, the conditional random field establishes a joint probability distribution model determined by the input sequence and the output sequence, and predicts the output sequence by maximizing the posterior probability in the joint probability distribution model, that is, under the conditions of the given input sequence, predict the optimal output sequence.

In the entity extraction task, the CRF can mark each word in the input text by determining whether the word is an entity (such as person name, place name, organization name, etc.), thus identifying the entity in the text. In contrast to rule-or pattern matching-based methods, the CRF does not require manual specification of rules or patterns, which can automatically learn the relationship between the input and output sequences, and then improve the accuracy and robustness of entity extraction. After entity extraction, the training data set is used to train the model and optimize the model parameters, so that the model can accurately predict the location and type of entities.

Entity extraction is used to extract the part of the corpus that is lexically identified as a noun and belongs to the class in the ontology construction mentioned above as an entity, and then to refine the property of the entity and the relationships between the entities. Example of entity extraction are shown on the left of Table 1.

**Table 1:** Selected entities and properties in the specification.

Entity	Property
High-rise buildings	Building height, number of plies
Plant	Height, area, fire resistance rating
Firewall	Fire resistance rating, anti-seismic capacity
Door	Height, thickness, fire resistance rating
Window	Height, width, bottom height
Railing	Height, spacing
Stair	Width, angle of tilt, fire resistance rating

Property extraction is to extract the information about the properties of the entities in the corpus, such as the thickness of the wall, the fire resistance level, the height of the staircase handrail, etc. The details are shown on the right side of Table 1.

**Figure 5:**
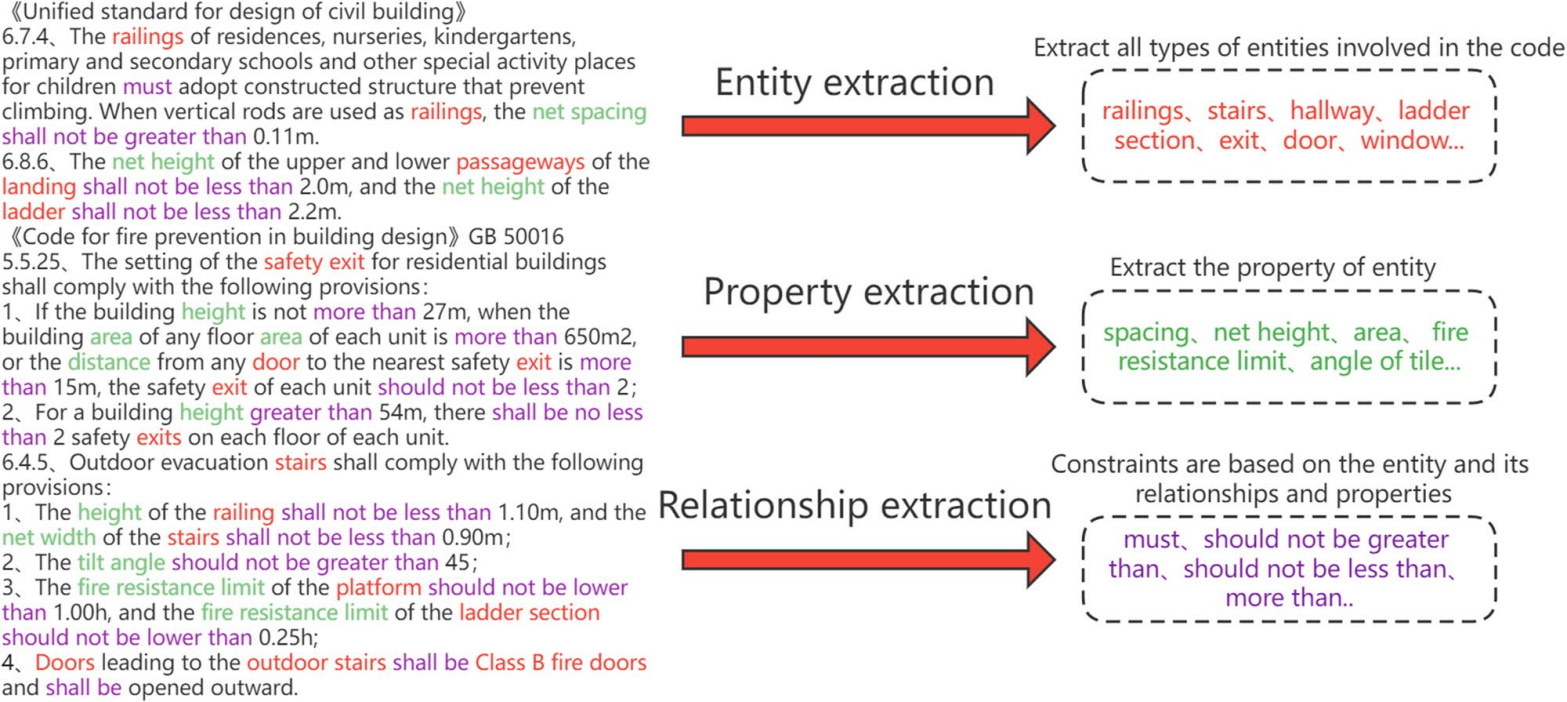
Schematic diagram of graph extraction.

Relationship extraction is to extract the association between entity–entity, entity-attribute, attribute-attribute or attribute-attribute value, so as to link the individual elements of entity and attribute together and finally form the form of triad. In this paper, all the relations existing in the specification are divided into two categories: space relations and size relations, which are shown in Table 2. A schematic representation of the extraction of the specification provisions is shown in Fig. 5.

**Table 2:** Relationship representation.

Space relationship	Size relationship
Contain	Should not be greater than
Above	Should not be less than
Below	Less than
Close to	More than
Connect	Should not be lower than
Separation	should be higher

In addition, this paper introduces a building norm relationship extraction method that uses attention mechanism to integrate sentence semantics. By analyzing and studying sentence semantic similarity and feature relationship, the sentence feature representation is constructed and input into the model. The key part of the model is the attention mechanism, it obtains word representation by analyzing the correlation level between words in sentences and modifying the weight coefficient matrix, as shown in formula 1:

1 $$Attention\,\left(Q,K,V\right)=softmax\left(\frac{{QK}^{T}}{\sqrt{{d}_{k}}}\right)V$$

where $Q,K,V$ are the word vector matrix, $\sqrt{{d}_{k}}$ is the Embedding scale. The weight coefficient representation is obtained by normalizing the ratio of the point multiplication between the $(Q,K)$ and the Embedding scale, and then multiplying by the matrix.

The semantic information of the sentence is obtained by analyzing the front and back information vector of the model. Finally, the attention mechanism assigns different weights to each word in the sentence, regards the low weight information as useless information and automatically ignores it, and only pays attention to the high weight information, so as to obtain the bytes that affect the extraction of entity relations.

#### Knowledge graph generation

**Figure 6:**
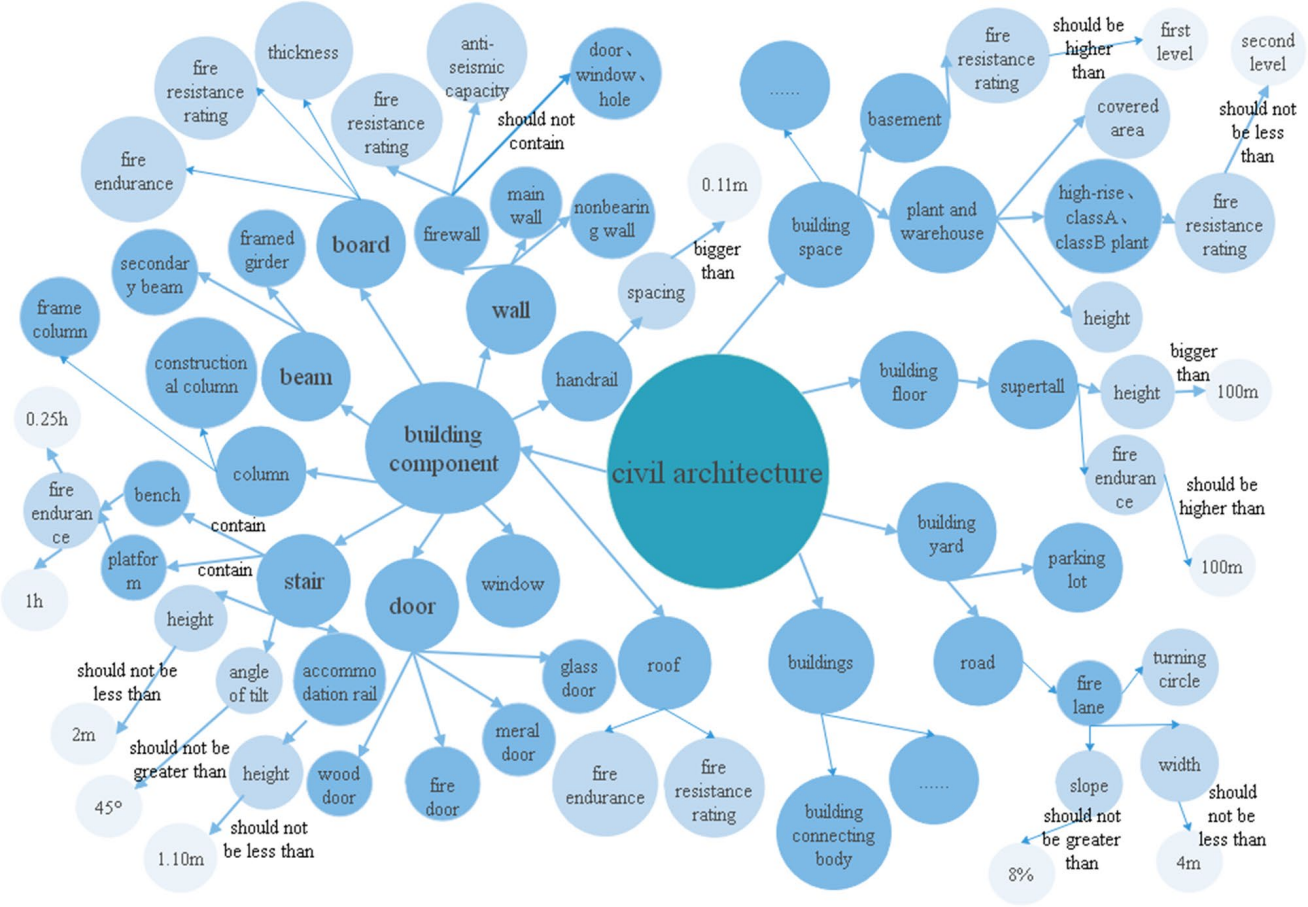
Partial knowledge graph.

After the schema and data layers of the knowledge map are constructed, they need to be stored in the database to form a human-readable triad; Neo4j is the current advanced graph database storage software, which saves the above entities, properties and relationships in the form of nodes and edges in Neo4j, store, query, and modify the data through the structure of the "graph". Therefore, in this paper, Neo4j is used to store the data. The data import process is divided into three steps: generation node, insertion relationship and configuration attribute, plus specific data to generate a computer-recognizable knowledge graph of civil building review specifications, as shown in Fig. 6. Blue balls indicate entities, such as wall, handrail, light balls indicates its properties, such as height is one of the properties of the stair, The text on the arrow indicates the relationship between them, such as stair contains bench and platform. Based on the diagram model, the knowledge graph of building review drawings is scalable and collaborative, and non-computer professionals can freely expand the specifications and store them, enhancing the practicality and applicability to future changes and developments of the specifications.

### BIM model analysis

With the development of building informatization, building information modeling has diverse data structure and multi-source heterogeneity, contains all the information of the whole life cycle of the building, with huge volume, wide sources and many involved parties. Not all BIM models can be directly used for automatic review; according to the preliminary research, the existing BIM modeling specifications of various professions cannot meet the review requirements^28,29^, which requires pre-processing of BIM models. In the building model information, there are already some physical and abstract concepts corresponding to building codes, such as the representation of physical entities, the concept of space, etc. There are also some concepts that do not explicitly exist or are hidden. Direct attributes such as height and thickness of walls can be automatically obtained from the BIM model exported file. For example, fire resistance class, water resistance class, seismic intensity and other parts not defined in the model by the designer can be extracted from the information in the form of a spreadsheet through the general design instructions. In addition, there are also indirect attributes such as window opening rate that need to be extracted through the design of the corresponding algorithm. Before exporting the data, the information requirements are defined according to the specification, and the information granularity (Level of Development, LOD) of the BIM model is determined. Then the model information is exported in IFC format, and since IFC files are long and redundant, the IFC redundancy deletion method proposed by Pan et al.^30^ The IFC format data belongs to the EXPRESS mode, convert it to the machine-readable IFCOWL form^31^, and then, together with external information, convert to the Resource Description Framework (RDF) format to be viewed in protégé, thus further clarifying the organizational format of the IFC file. Using the final resulting resource global description structure as an ontology instance avoids formatting differences due to different data sources.

### Rule enforcement

In terms of geometric computation and data consistency correspondence, the mapping between the model and the rule code is performed through a modular algorithm design that allows different modules to be used overlappingly, and the mapping rules alleviate the linguistic ambiguity between the information; the review process of the BIM model is realized through consistency comparison^32,33^. Since the object of review in the code review process presented in the knowledge graph is "entities" rather than the BIM model itself, it is necessary to bind the derived component information in the model to the entities in the knowledge graph for compliance review, and the logic of automatic code consistency checking involves two parts, i.e., mapping rules and checking rules. To achieve complex mapping, data query and external programming are necessary. Mapping rules are used to link the ontology of mapping review rules and design model instances one by one, which mitigate the semantic ambiguity between design information and code information. Mapping rules can be divided into entity mapping and attribute mapping. The term mapping rules are mainly used to deal with the possible semantic ambiguities of entities and attributes, this process requires the user to provide sufficient information or mark the data in advance. The check rules are a set of programs built from the review rules, and the check rule program is executed after mapping to traverse the review list and execute its corresponding check commands for different entities. Check rules are explained by the compiled code, and can generally fall into four categories: class 1-rules that require a single or a small number of explicit data; class 2-rules that require simple derived attribute values; class 3-rules that require extended data structure; class 4-rules that require a “proof of solution.” Check that the properties of an entity belong to the class 1, such as the handrail height. Calculating whether the window-to-floor ratio of a room meets the requirements belongs to the class 2. The class 3 can be applied to check the non-spatial or non-numeric relationship between two entities, such as checking the number of entities. The class 4 can be used to query solutions, such as finding a floor with only one entrance and exit. This study makes use of SPARQL queries and external programming to specify the mapping and checking process. SPARQL is a query language and data acquisition protocol developed for RDF, providing a complete set of analytical query operations for data. The increasing number of Knowledge Graphs (KGs) available today calls for powerful query languages. SPARQL provides a specific graph traversal syntax for the data that can be viewed as a graph.

### System design

In order to realize the automatic review system for the BIM model, in this paper, the B/S (Browser/Server) grid structure design is selected to develop the BIM automatic review system. In this structure, the server side carries the main logical things, and does not display the complex logic of things on the browser side. The user’s working interface is mainly realized through the browser side. This structure simplifies the computer load on the client side, reduces the use cost of users, and is more convenient, fast and efficient for the software of the automatic review system.

In this system, the bottom-up level is divided into four levels, the data layer, the service layer, the application layer and the access layer. The data layer mainly includes the database of the automatic review system, such as BIM graph data and knowledge graph data, ontology data, etc., is the basis of the system implementation. The service layer is the core layer of the system, which is used to connect the underlying data and the upper application, to realize the data call. In the application layer, the functional modules are mainly implemented, mainly including the 3 D front-end display of the BIM model. At the access layer, users can interact with functions through PC, mobile and other devices.

After performing a reasoning process based on mapping rules and checking rules on the merged ontology based on specification review rules, the results of the checklist are combined with the specification. The results of the system output can be divided into three categories: (1) pass, i.e., the component is a specification-compliant entity; (2) fail, i.e., the component does not meet the specification requirements and needs to redefine its characteristics, in which case the system will issue a review report stating non-compliant component and the corresponding original specification content; and (3) warning, which indicates the need for manual re-checking.

## Case studies

### Preparation for implementation

**Figure 7:**
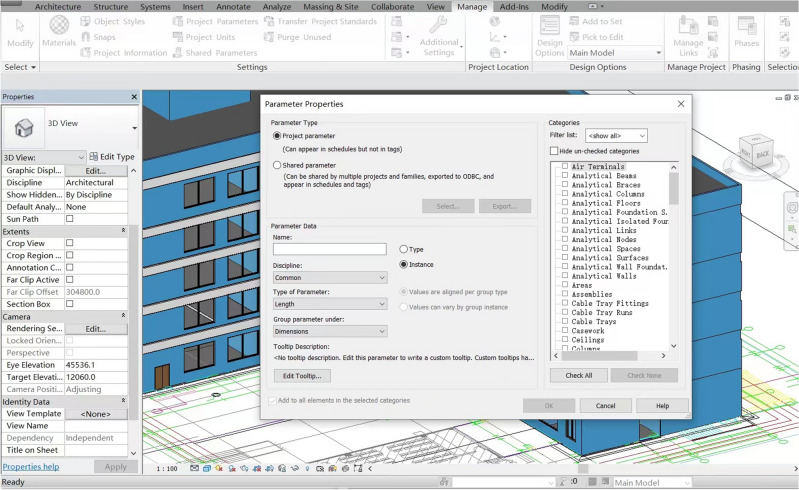
Model pre-processing.

**Figure 8:**
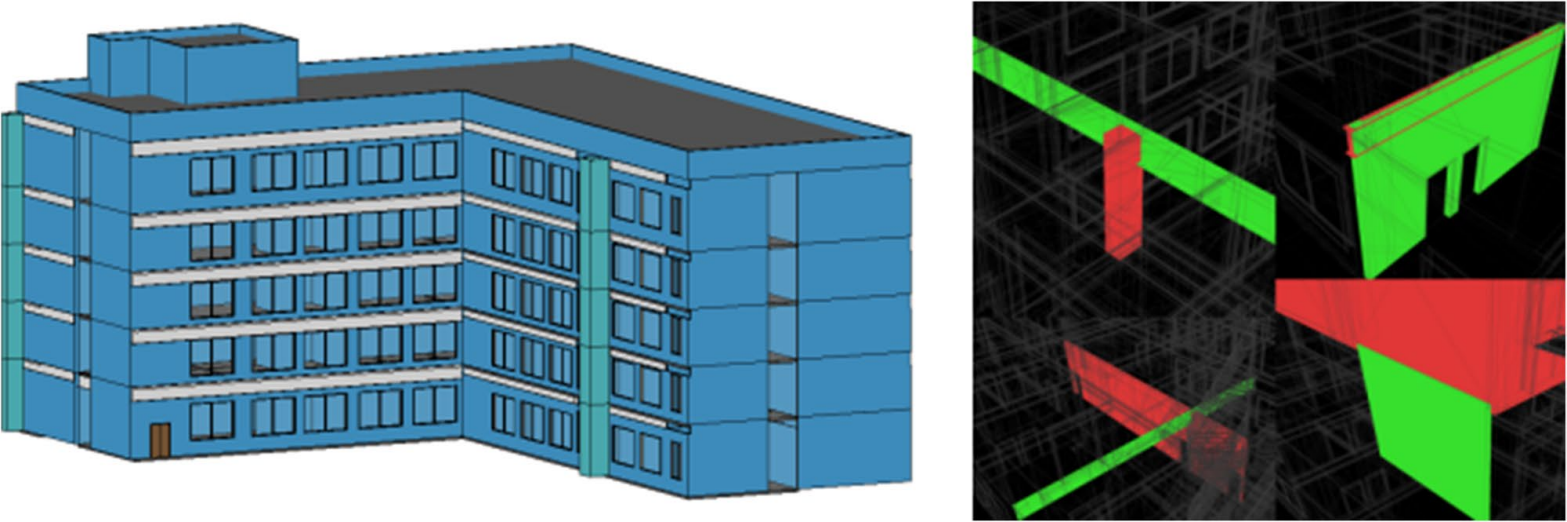
Model and collision check.

**Figure 9:**
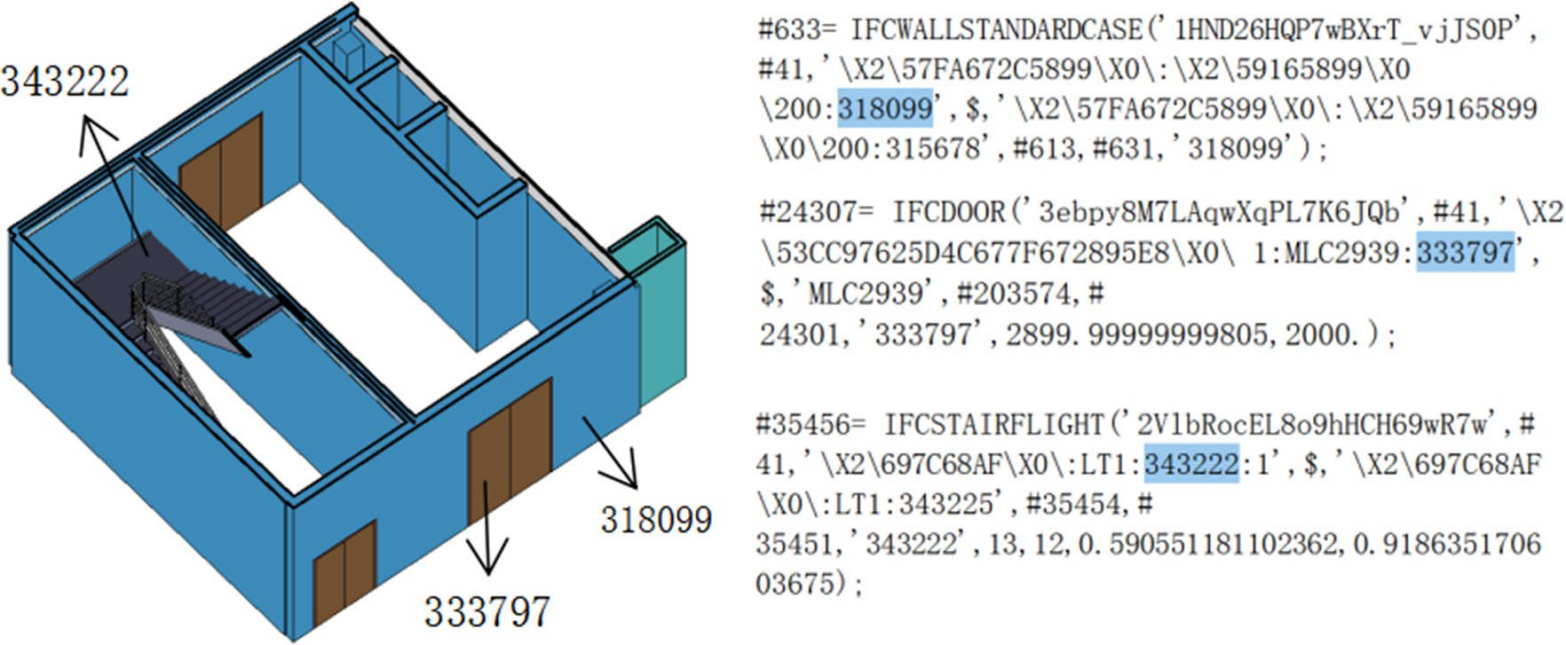
IFC document.

**Figure 10:**
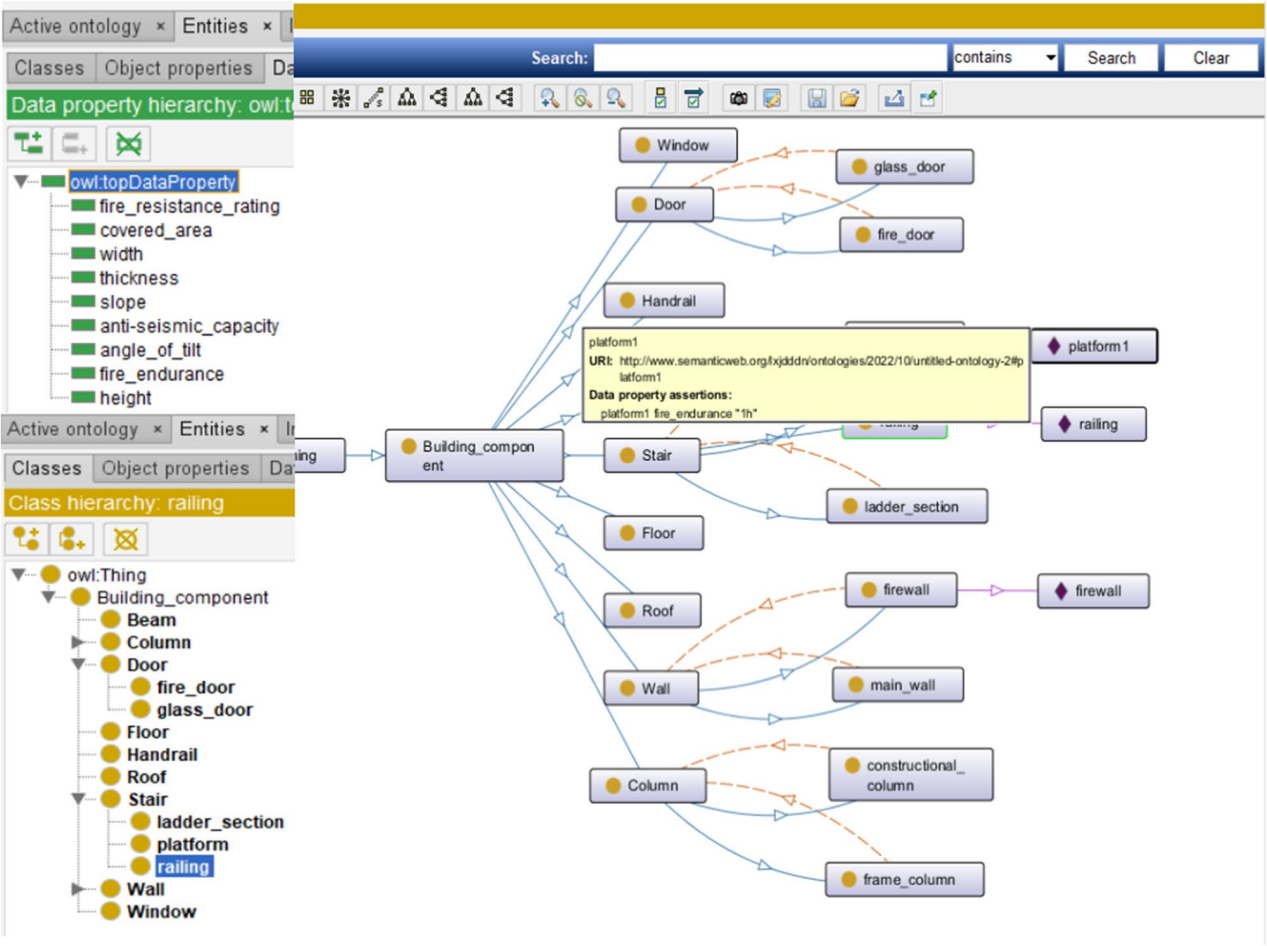
BIM information.

In order to verify the feasibility of this study, this paper uses revit software for modeling, pre-processing the model, defining the project parameters in detail, as shown in Fig. 7, defining the functions of each room and the corresponding property relationships of the components, which are checked and corrected by collision analysis, as shown in Fig. 8: BIM model of a building and collision check. The data is exported in IFC format, as shown in Fig. 9, and the building components are represented as numerical codes in the IFC file. In the next step, the IFC file is converted into an IFCOWL file using the IFC to RDF converter, which converts the file into a standard format that is easier to read by the computer using the method mentioned in Sect. Construction of the knowledge graph, and also adds some additional information to the model ontology, such as an overview of the building model, the type of structure, building function, seismic rating, etc., which is usually included in the general description of the building. The final result is a resource description framework database containing global information about the building model. As shown in Fig. 10 in protégé you can view the model related entities, attributes and relationships, and finally get the building model data information in RDF format.

In this study, selected provisions from the 《Uniform Standard for Civil Building Design》 and the 《Code for Fire Protection in Building Design》 are used to check the compliance of the test cases, selected regulations from the knowledge graph generated in Sect. Knowledge graph generation that are applicable to this case. The automatic review system designed and developed in this paper has built-in specification knowledge ontology and structured rule logic expressions required in the Specification. That is, the knowledge graph is saved as a graph database, and the specification standard is automatically called to review the design of the BIM model during the automatic review process. In order to verify the accuracy of the review results, two non-compliances of the model that did not match the selected specification regulations were preset in advance before starting the review. After completing the establishment of the specification database and the engineering information database, the matching analysis of the engineering information data files based on SPARQL review rules needs to be performed in conjunction with an expert system during the rule inference review phase. As shown in Table 3, the ontology mapping is proposed to mediate the ontology heterogeneities on terminologies and descriptions between the two ontologies and enrich the merged ontology semantics in this paper. The review rule query statement is shown in Table 4, which is traversed against a set of subject-predicate-object triples for the database. For example, the first judgment whether the height of the stair handrail is not less than 1.10 m, the rules first find the entity type of the stair handrail, then determine the attribute type to be reviewed, and finally make a numerical judgment to obtain the result.

**Table 3:** Example of mapping rules.

Number	Mapping entity	SPARQL mapping rule
1	Door	(?x rdf:type :IfcDoor) - > (?x rdf: type Code:Door)
2	Wall	(?x rdf:type :IfcWall) - > (?x rdf: type Code:Wall)
3	Stair	(?x rdf:type :IfcStair) - > (?x rdf: type Code:Stair)

**Table 4:** Examples of review rules.

Number	Rule	SPARQL investigate rule
1	The height of handrail should not be less than 1.10 m	SELECT? a? b WHERE { ? a rdf: type ont: handrail.? a ont: height? b filter ( ? b > 1.10) }
2	The fire resistance of ladder section is greater than 0.25 h	SELECT? a? b WHERE { ? a rdf: type ont: ladder section.? a ont: fire resistance? b filter ( ? b > 0.25) }
3	The area of kitchen is not less than 6 mm^2^	SELECT? a? b WHERE { ? a rdf: type ont: kitchen.? a ont: area? b filter ( ? b < 6.00) }

### Implementation results

The review of each prototype component by the above system resulted in the following.

1. Fail: the height of the railing handrail shall not be less than 1.10 m, The instance railing does not meet the provisions of Article 6.6.1 of the 《General Code for Civil Construction》GB 55031-2022.
2. Fail: the fire resistance limit of the stairway platform shall not be less than 1 h. The instance platform1 does not meet the provisions of Article 6.4.5 of 《Code for fire prevention in building design》GB 50016.

The rest of the sections pass the compliance review, and users can preview the review results online through the system or download a report of the review results as a basis for model modifications. The above results are consistent with the two default non-compliance, demonstrating the effectiveness of the automated code consistency checking system.

### Expert review

To demonstrate the accuracy of this study, experts in the industry are invited to manually review the example drawings and obtain the following review comments:

The design depth of the construction drawing of this project basically meets the requirements of the construction engineering code, but there are still errors and omissions in the drawing, and some parts do not meet the code, which should be reviewed after supplement and modification. The main engineering problems are as follows:

1. The fire resistance limit of the platform is 0.25 h, which does not meet the provisions of Article 6.4.5 of 《Code for fire prevention in building design》GB 50016. Please modify and improve it.
2. The handrail height of vertical rod parts is 1.00 m, which does not meet the provisions of Article 6.6.1 of the《General Code for Civil Construction》GB 55031-2022. Please modify and improve it.

The above results can show the accuracy of the method, but from the perspective of time and efficiency, the automatic code compliance checking system has significant advantages. Manual inspection is observed by human eye, which is time-consuming and easy to miss, automatic code checking can scan quickly to detect potential problems. The accuracy of manual inspection is affected by the professional level of personnel, and the labor cost is high, the cost of using computer programs is relatively lower.

### Comparative analysis

In this paper, four BIM engineering projects of different building types are selected to evaluate the proposed method from two dimensions of time efficiency and precision. As shown in Table 5, $t$ represents the time taken to complete the model review, $P$ represents the accuracy rate of the system component review. The calculation method is shown in Eq. 2:

**Table 5:** BIM Model Test Results.

Experiment number	Project name	$t (s)$	$P$(%)
1	Classroom building	8.847	96.81
2	Subway station	4.526	97.26
3	Petrol station	3.358	98.17
4	Apartment	7.031	97.33

2 $$P=\frac{\text{Number of components correctly detected in the test}}{\text{Number of all components detected in the test}}\times 100\%$$

According to the above analysis, the proposed system can complete the common model review in a very short time, the accuracy of its review has also reached more than 96%. Compared with the traditional manual drawing review, the drawing review system developed in this paper has greatly improved in timeliness and review accuracy.

## Discussion

This paper focuses on the study of an automated compliance checking system for BIM models, and for several steps of code checking, this system is divided into the following modules: an ontology development module, which aims to establish a specification text checking database. An information input module, which is used to input information about the model to be reviewed and to transform BIM model component information and information external to the project into a data interpretation structure that can be understood by a computer. A knowledge graph module, which presents the specification database in the form of a knowledge graph to facilitate the transformation into inspection rules by external programming. The mapping and review module, which serves as an intermediary between the model ontology and the rule ontology to ensure the subsequent review commands. And the result output module, which outputs the inspection report after the inspection.

Compared with previous studies, the contribution of this study is the application of natural language processing techniques to transform specifications into checking rules in the form of knowledge graphs. In addition, the semantic information of the building model is enriched and the semantic contradictions are alleviated by using mapping relations. Relying on external programming to solve the problem of geometric calculation of building models, and to obtain more comprehensive building information.

## Conclusion

Automated compliance checking is a key item in the current reform of the construction engineering industry, who has changed the previous mode of relying on manual review, liberating manpower while making the review more accurate and efficient. In this paper, we propose an automatic code compliance system based on BIM and knowledge graph, which uses semantic web technology to convert specification provisions into knowledge graph to form specification knowledge base ontology, the integration of BIM model information as well as external and indirect information belongs to the information input side of the system, the mapping rules and inspection procedures rely on external programming to realize, the terms at the information input side are identified by the computer and the knowledge base ontology side entity link is formed, and then the knowledge mapping-based rule checking command is executed, and finally the checking report is generated based on the checking results. Based on the above approach, a case study was conducted using a BIM model. The main findings of this study are as follows.

1. Rules in the form of knowledge graphs presented in the system repository are easier for system operators to understand and identify changes, saving operational memory and runtime while providing greater agility in querying data.
2. Mapping rules can effectively resolve semantic ambiguities between IFCOWL documents and canonical texts.
3. The modular algorithm design can effectively solve multi-modal multifaceted inspection commands and face complex inspection tasks by overlaying multi-modal algorithms.

If this study is to be truly applied, on the one hand, the use of BIM in the design phase must be promoted, and the introduction of a standardized document for BIM design is essential. In the process of BIM model design, the needs of later graph review should be taken into account, such as adding rich semantic information to the model. In addition, in the future, the BIM model design software can be considered to establish synergy with the review software, which reduces the possible errors and omissions in the process of data transfer. This study considers mainly the review of BIM construction models before construction, and the review of BIM progress management, cost and other information during construction is still a part to be expanded. In addition, automatic review is emerging and still faces great challenges in model semantic expansion, complex engineering knowledge representation learning, performance-based design review, algorithm robustness and transparency.

## Data Availability

The datasets used and/or analysed during the current study available from the corresponding author on reasonable request.
